# Overexpression of METTL3 associated with the metabolic status on ^18^F-FDG PET/CT in patients with Esophageal Carcinoma

**DOI:** 10.7150/jca.44754

**Published:** 2020-06-07

**Authors:** Xu-Sheng Liu, Ling-Ling Yuan, Yan Gao, Lu-Meng Zhou, Jian-Wei Yang, Zhi-Jun Pei

**Affiliations:** 1 Department of Nuclear Medicine and Institute of Anesthesiology and Pain, Taihe Hospital, Hubei University of Medicine, Shiyan, 44200, China.; 2 Department of Pathology, Taihe Hospital, Hubei University of Medicine, Shiyan, 442000, China.; 3 Hubei Key Laboratory of WudangLocal Chinese Medicine Research, Shiyan, 442000, China.; 4 Hubei Key Laboratory of Embryonic Stem Cell Research, Shiyan, 442000, China.

**Keywords:** Esophageal carcinoma, methyltransferase 3, ^18^F-FDG, glucose transporter 1, hexokinase 2

## Abstract

**Background:** To investigate the expression of methyltransferase 3 (METTL3) and its relationship with ^18^F-FDG uptake in patients with esophageal carcinoma (ESCA).

**Materials and methods:** This study analyzed the expression of METTL3 in ESCA and its relationship with clinicopathological features by The Cancer Genome Atlas (TCGA) database. Immunohistochemical staining was performed on 57 tumor tissues of ESCA patients who underwent PET/CT scan before surgery to evaluate the expression of METTL3, glucose transporter 1 (GLUT1), and hexokinase 2 (HK2) in tumor tissues and peritumoral tissues. Analyze the relationship between SUVmax with METTL3, HK2, and GLUT1 expression.

**Results:** The expression of METTL3, GLUT1, and HK2 was significantly increased in ESCA tissues compared with normal tissues (*p <* 0.001). The expression of METTL3 was correlated with tumor size and histological differentiation (*p <* 0.05), and there was no significant difference between age, sex, pathological types, tumor staging, or lymph node metastasis (*p* > 0.05). The SUVmax was significantly higher in tumors with high METTL3 expression (17.822±6.249) compared to low METTL3 expression (9.573±5.082) (*p <* 0.001). There was a positive correlation between the SUVmax and METTL3 expression in ESCA (*r^2^* = 0.647, *p <* 0.001). Multivariate analysis confirmed the association between SUVmax and METTL3 expression (*p <* 0.05). GLUT1 and HK2 expression in ESCA was positively correlated with ^18^F-FDG uptake and METTL3 status (*p <* 0.001).

**Conclusions:** The high expression of METTL3 is related to the high SUVmax in ESCA, and METTL3 may increase ^18^F-FDG uptake by regulating GLUT1 and HK2.

## Introduction

Esophageal carcinoma (ESCA) is one of the six most common malignant tumors in humans, and it is the third most common cancer in China 1, 90% of which are esophageal squamous cell carcinoma 2. Despite some progress in disease diagnosis and cancer treatment 3-5, the prognosis of patients with advanced ESCA is still very poor, and the survival rate of patients within 5 years is only 20%-30% 6. Traditionally, endoscopic examination and mucous membrane biopsies have been widely used in early ESCA detection 7-9, but invasive examination limits its application in asymptomatic population screening. Therefore, the development of a noninvasive biomarker for early diagnosis of ESCA is essential for improving patient survival.

N6-methyladenine (m6A) is a methylation modification that occurs at the 6th nitrogen atom of adenine and is the most common RNA modification in mammalian mRNA 10-12. Methyltransferase 3 (METTL3) and methyltransferase 14 are methyltransferases responsible for m6A modified mRNA transcripts, while METTL3 is a core component of m6A methyltransferase 13-15. Recently, some studies have shown that METTL3 is overexpressed in many cancer tissues and plays an important role in occurrence and development 16-18. Therefore, studying the efficacy of METTL3 in ESCA and predicting the extent of METTL3 expression is critical for METTL3 targeted therapy.

Positron emission tomography/computed tomography (PET/CT) is a new molecular imaging technique 19-21. ^18^F-fluorodeoxyglucose (^18^F-FDG) PET/CT imaging can estimate the state of glucose metabolism in tumors 22-26. A high standard uptake value (SUVmax) has been shown to correlate with the malignancy of multiple tumors 27-29. ^18^F-FDG PET imaging is based on the high metabolic rate of glucose in tumor cells, and this mechanism of increase in glucose consumption and aerobic glycolysis in tumor cells is called the Warburg effect 30, 31. Glucose transporter 1 (GLUT1) and hexokinase 2 (HK2) are the major proteins regulating glucose transport and transformation, respectively, and are the rate-limiting enzymes of the Warburg effect, which are closely related to the rate of ^18^F-FDG uptake by ESCA 32, 33. Studies have shown that GLUT1 and HK2 are highly expressed in various tumors including ESCA 32-36, but there is no research on the relationship between GLUT1 and HK2 with METTL3 expression.

This study analyzed the relationship between the expression of METTL3 in ESCA and clinicopathological features in the The Cancer Genome Atlas (TCGA) database and verified it by immunohistochemical analysis. At the same time, we investigated the relationship between ^18^F-FDG uptake and METTL3 expression levels to verify whether ^18^F-FDG uptake can be used to predict the status of METTL3 in ESCA patients. Through analysis, we determined whether the expression levels of GLUT1 and HK2 were related to ^18^F-FDG uptake and assessed the association of METTL3 with GLUT1 and HK2 in ESCA. Our results indicate that the high expression of METTL3 is related to the high SUVmax in ESCA, and that ^18^F-FDG PET may help determine the therapeutic strategy for ESCA patients by predicting tumor response to METTL3-targeted therapies.

## Materials

### Study population

This present study included 57 consecutive ESCA patients who underwent baseline ^18^F-FDG PET/CT and surgical resection from January 2016 to December 2017. The selection criteria are as follows: (a) pathologically confirmed ESCA, (b) no biopsy, chemotherapy or radiation therapy before PET / CT imaging, (c) the time interval between PET / CT imaging and surgery is less than 1 month, (d) complete case information includes age, gender, pathological type, tumor size, histological differentiation, tumor staging, and lymph node metastasis. Our retrospective study was approved by the Institutional Review Board of the Hubei University of Medicine-affiliated Taihe Hospital, and the requirement to obtain informed consent was waived.

### PET/CT imaging and analysis

Images were acquired using a combined PET/CT device (Biograph mCT; Siemens). All patients received an intravenous injection of 0.1mCi/kg (3.7 MBq/kg) of ^18^F-FDG after having fasted for at least 6h. The mean uptake time was 50 min. PET images were iteratively reconstructed, and CT data were used for attenuation correction. For semi-quantitative analysis of ^18^F-FDG uptake, all PET/CT images were transferred to a PACS workstation (Power Imager, mozihealthcare, Beijing) and the image was post-processed, the region of interest (ROI) was manually defined on the transaxial tomograms and the SUVmax was automatically calculated by the software. Two experienced nuclear medicine physicians evaluated the PET images. SUV was calculated as follows:


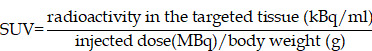


### UALCAN

UALCAN (http://ualcan.path.uab.edu) is a newly developed web portal based on level 3 RNA-seq and clinical data from 31 cancer types in TCGA database 37. With UALCAN, we evaluated the mRNA expression level and methylation level of METTL3 in ESCA and normal Esophagus samples. And it analyzed relative expression of the METTL3 gene in various tumor subgroups based on gender, age, tumor histology, tumor grade, individual cancer stages and nodal metastasis status or other clinic pathologic features. The difference of transcriptional expression was compared by student's t-test and *p <* 0.05 was considered as statically significant.

### Immunohistochemistry and analysis

All 57 ESCA patient specimens were fixed in formalin and embedded in paraffin. Paraffin sections having a thickness of 4 μm were dewaxed by xylene, gradient alcohol, and distilled water. Tissue sections were heated in a microwave oven for 10 minutes in 100X Tris-EDTA Buffer (pH 9.0) (Abcam, USA) and then incubated with 3% hydrogen peroxide solution (zsbio, Beijing) for 10 minutes to inhibit endogenous peroxide activity. The sections were blocked with non-immune serum for 15 min and then incubated overnight with a METTL3 rabbit anti-human monoclonal antibody (1:200, Abcam, USA), GLUT1 mouse anti-human monoclonal antibody (1:200, Abcam, USA) and HK2 mouse anti-human monoclonal antibody (1:100, Abcam, USA) in a 4 °C refrigerator. After rewarming for 15 min at room temperature the next day, it was washed 3 times with Tris-Buffered Saline Tween-20 (TBST) for 5 min each time. Goat anti-mouse (1:300, Abcam, USA) and Donkey anti-rabbit (1:500, Abcam, USA) second antibody were added to the sections and incubated for 1 h at room temperature, followed by washing 3 times with TBST for 5 min each time. Each section was added with DAB reagent to develop color and then counterstained with hematoxylin. Next, the sections were dehydrated in gradient alcohol and xylene and finally sealed with neutral gum.

The material was evaluated with the use of a light microscope using ×40 lenses and utilized the IHC Profiler plugin of ImageJ software to quantify the staining intensity 38. Of a single slide at least 10 random images were taken. In the IHC profiler, four types of scoring were followed based on the pixel count of staining intensity: high positive (score 3), positive (score 2), low positive (score 1), and negative (score 0). We took an average score of 10 random images and categorized the METTL3, GLUT1, and HK2 expression as follows: 0-1.9, low expression; 2-3, high expression.

### Statistical analyses

The data were presented as mean ± standard deviation (SD). The expression of METTL3, GLUT1, and HK2 in tumor tissues and peritumoral tissues and the significance of SUVmax between low expression group and high expression group were analyzed by independent sample T-test. Assessment of the association between METTL3 expression and various clinical parameters in ESCA patients using the chi-square test and the relationship between METTL3 with GLUT1 and HK2 expression was assessed. The relationships between SUVmax with METTL3, GLUT1 and HK2 expression were analyzed using Pearson correlation coefficients. The receiver operating characteristic (ROC) curve was used to assess the optimal value of SUVmax for predicting METTL3 expression. Multivariate analysis was used to analyze factors associated with METTL3 expression. *p <* 0.05 was considered significantly different. All statistical analyses were conducted with SPSS software (SPSS, version 19.0).

## Results

### Patient characteristics

Table 1 shows the clinicopathological features of 57 patients with ESCA, including 43 males and 14 females. The mean patient age was 57 years and the range was 41-77 years. There were 51 cases of esophageal squamous cell carcinoma and 6 cases of adenocarcinoma. The mean tumor size was 3.95 cm and the range was 1.10-9.0 cm. The mean primary tumor SUVmax was 14.20 and the range was 3.12-30.25. Lymph node metastasis was detected in 30 patients. There were 25 patients with poorly differentiated tumors and 32 patients with moderately-well differentiation.

### Analyzing METTL3 from TCGA database generally increases the expression in ESCA

**Figure 1:**
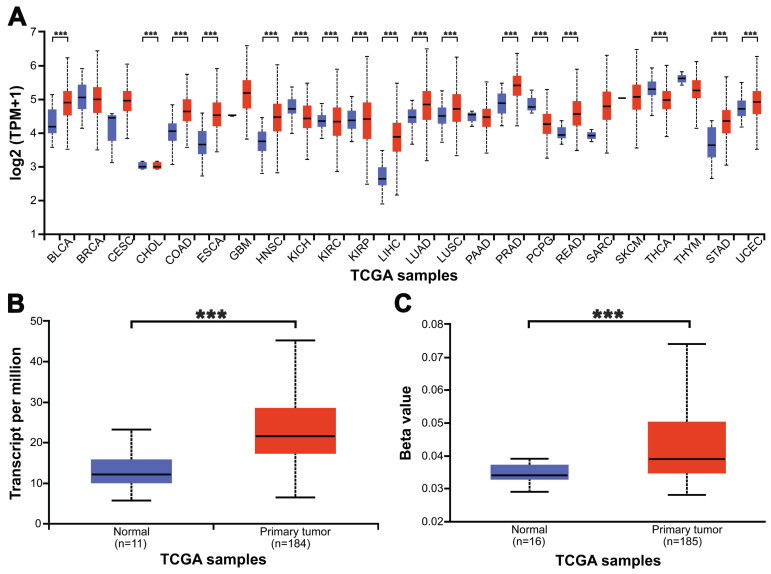
The expression level of METTL3 in ESCA and adjacent normal esophageal tissues from the TCGA database.** (A)** The expression of METTL3 in 24 types of tumor tissues and paired normal tissues. **(B)** The expression of METTL3 was elevated in ESCA compared to normal esophageal tissues. **(C)** Comparison of the promoter methylation level of METTL3 in 16 cases normal Esophageal and 185 cases tumor tissues. ***p < 0.001.

We used UALCAN to check the expression level of METTL3 in 24 kinds of tumors and paired normal tissues in the TCGA database (Fig. 1A, *p <* 0.001). The results showed that METTL3 was highly expressed in 17 tumors including bladder urothelial carcinoma (BLCA), extrahepatic cholangiocarcinoma (CHOL) and colon adenocarcinoma (COAD). Low expression was found in four kinds of tumors, such as kidney renal clear cell carcinoma (KIRC) and thyroid carcinoma (THCA). To confirm the role of METTL3 in the occurrence and development of ESCA, we used the TCGA database to analyze the transcription levels of METTL3 in tissues, including 184 ESCA tissues and 11 normal esophageal tissues. Compared with normal esophageal tissue, the transcription level of METTL3 in ESCA tissues is significantly higher (Fig. 1B, *p <* 0.001). As shown in Figure 1C, the methylation level of METTL3 in esophageal tumors was significantly higher than that in normal esophageal tissues (*p <* 0.001). These results indicate that METTL3 may play an important role in the occurrence and development of ESCA.

### Relationship between the mRNA levels of METTL3 and the clinicopathological parameters of ESCA patients

**Figure 2:**
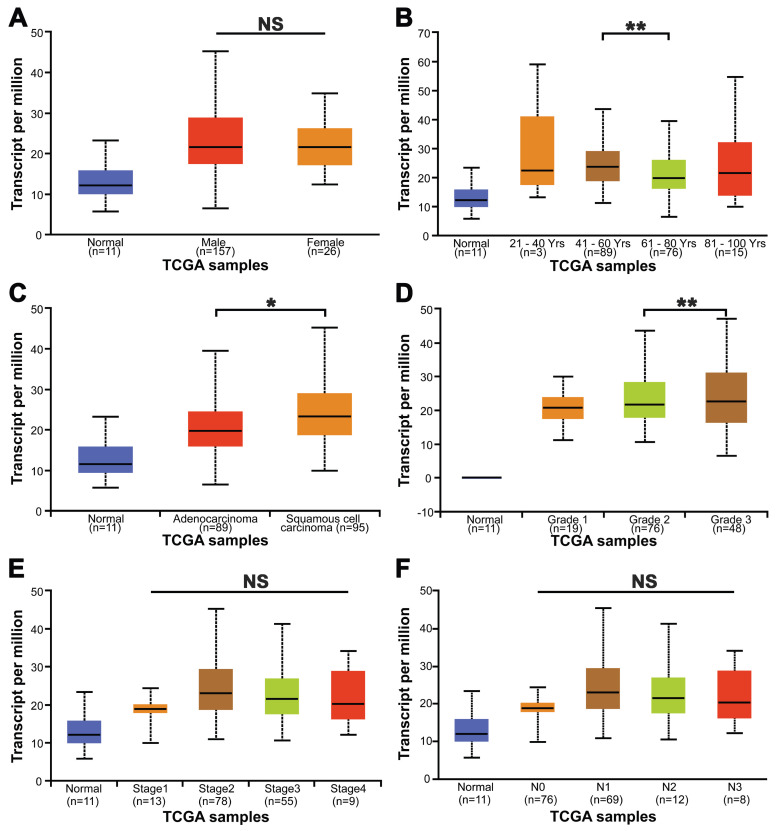
Relationship between the mRNA levels of METTL3 and the clinicopathological parameters of ESCA patients. The relationship between METTL3 relative transcriptional expression levels and patients' gender (**A**), age (**B**), tumor histology (**C**), tumor grade (**D**), tumor stage (**E**) and lymph node metastasis (**F**). *p < 0.05, **p < 0.01.

The relationship between the relative transcription level of METTL3 and the clinicopathological parameters of ESCA patients was analyzed by UALCAN (Fig. 2). The analysis showed that the relative transcription level of METTL3 was not associated with ESCA patients' gender (Fig. 2A), tumor stage (Fig. 2E) and lymph node metastasis (Fig. 2F) (*p* > 0.05). Although METTL3 mRNA expression was higher in patients aged 41-60 years than in those aged 61-80 years (*p* = 0.008), there was no difference in METTL3 mRNA expression between patients aged 21-40 years, 61-80 years and 81-100 years old (Fig. 2B). Therefore, it can be considered that there is no correlation between METTL3 expression level and age. The expression of METTL3 mRNA in esophageal squamous cell carcinoma and esophageal adenocarcinoma was significantly different (Fig. 2C, *p* = 0.044). METTL3 mRNA expression was significantly different between grade 2 and grade 3 in ESCA tumor grades (Fig. 2D, *p* = 0.003). There was no difference between grade 1 and other groups, possibly due to the small number of samples.

### Expression of METTL3 in ESCA patients and its relationship with clinical features

**Figure 3:**
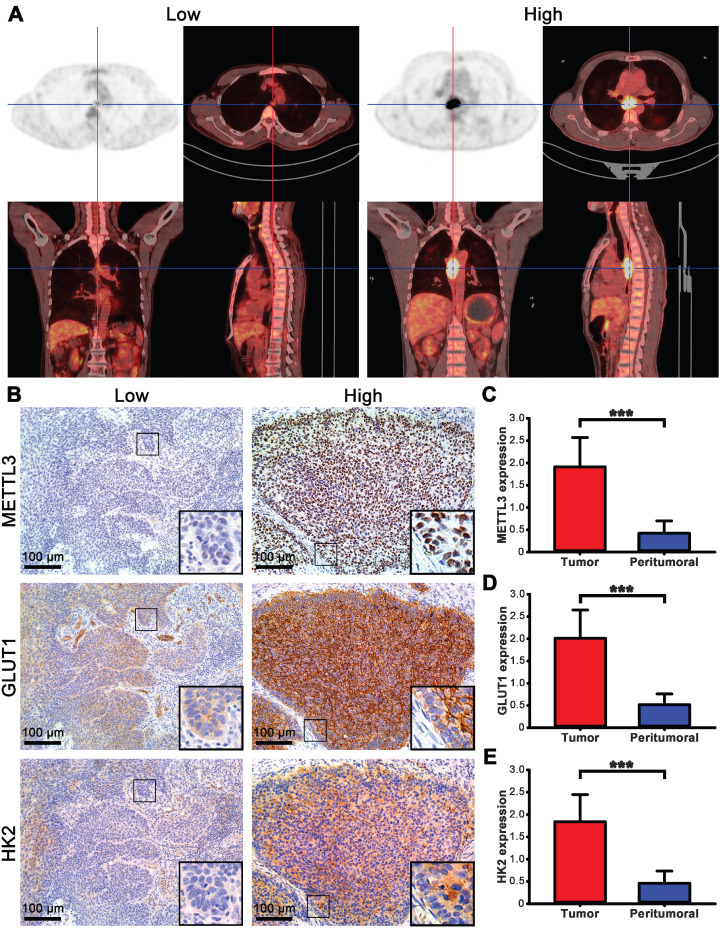
The expression of METTL3 in ESCA patients. **(A)** PET/CT imaging of patients with ESCA with different SUVmax uptake values. Left picture SUVmax=3.61, right picture SUVmax=22.08. **(B)** Immunohistochemical staining of METTL3, GLUT1, and HK2 in ESCA patients with different SUVmax uptake values. **(C)** The mean METTL3 immunohistochemical staining score in ESCA tissue (1.912±0.658) was significantly higher than that of matched peritumoral tissue (0.425±0.278). **(D)** The mean GLUT1 immunohistochemical staining score in ESCA tissue (2.011±0.637) was significantly higher than that of matched peritumoral tissue (0.519±0.242). **(E)** The mean HK2 immunohistochemical staining score in ESCA tissue (1.839±0.610) was significantly higher than that of matched peritumoral tissue (0.460±0.275). *** p < 0.001.

The FDG uptake value of lesions in all ESCA patients was high or low, but it is increased compared with normal tissues (Fig. 3A). To understand the role of METTL3 in ESCA, we compared METTL3, GLUT1 and HK2 expression between tumor and matched normal tissue specimens from the 57 patients. In the tumor cells, METTL3 expression was mainly detected in the nucleus, GLUT1 and HK2 were mainly detected in the cytoplasm, but GLUT1 was also detected in the cell membrane (Fig. 3B). Positive METTL3 expression was observed in 93.0% (53/57) of the tumor and 8.7% (5/57) of peritumoral tissues (Table 2). The mean METTL3, GLUT1, HK2 immunohistochemical staining score in the tumor tissues was significantly higher than that in the peritumoral tissues (Figs. 3C-E, *p <* 0.001). Based on the immunohistochemical analysis, the ESCA patients were classified into two groups: those with high (n=32) and low (n=25) METTL3 expression. The relationship between METTL3 expression and various patient clinicopathological parameters is illustrated in Table 3. METTL3 expression was correlated with tumor size and histological differentiation (*p <* 0.05). However, there were no significant differences between METTL3 expression and age, sex, pathological types, tumor staging, or Lymph node metastasis (*p* > 0.05).

### SUVmax is positively correlated with METTL3 expression

**Figure 4:**
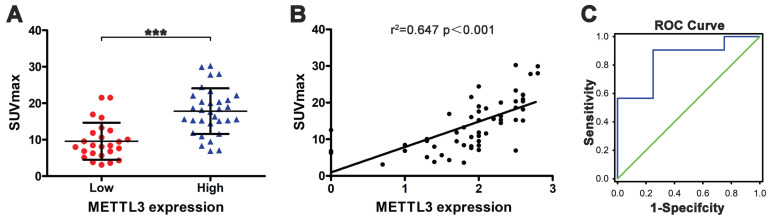
The relationship between SUVmax and METTL3 expression in ESCA.** (A)** The SUVmax of tumors with high METTL3 expression (17.822±6.249) was significantly higher compared to low METTL3 expression (9.573±5.082) (*p* < 0.001). **(B)** The SUVmax and METTL3 expression levels are positively correlated (*r^2^*= 0.647, *p* < 0.001). **(C)** Receiver operating characteristic curve analysis showed that when the SUVmax cutoff value was 6.81, the area under the curve was 0.844±0.090 (95%CI = 0.667-1.020, *p* = 0.023). Sensitivity and specificity for the prediction of METTL3 expression were 90.6% and 75.0%, respectively.

Next, we evaluated the relationship between SUVmax and METTL3 expression in ESCA. The SUVmax of tumors with high METTL3 expression was significantly higher compared to low METTL3 expression (17.822±6.249 vs 9.573±5.082) (Fig. 4A, *p <* 0.001). There was a positive correlation between the SUVmax and METTL3 expression levels in ESCA (Fig. 4B, *r^2^* = 0.647, *p <* 0.001). We determined that the SUVmax threshold was used to distinguish patients with positive and negative METTL3 expression. The ROC curve analysis showed that when the SUVmax cutoff value was 6.81, the area under the curve was 0.844±0.090 (95%CI = 0.667-1.020, *p* = 0.023). Sensitivity and specificity for the prediction of METTL3 expression were 90.6% and 75.0%, respectively (Fig. 4C). These results indicate that SUVmax in ESCA patients can be used to predict METTL3 expression status at tumor sites. Multivariate analysis demonstrated a correlation between SUVmax and METTL3 expression (Table 4, *p* = 0.001).

### Association of 18F-FDG uptake and METTL3 expression with GLUT1 and HK2 expression

**Figure 5:**
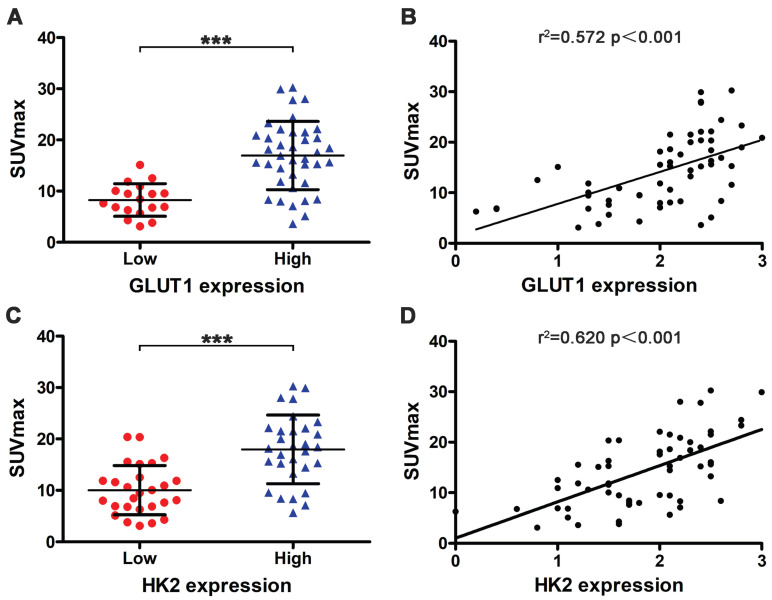
Relationship between SUVmax with GLUT1 and HK2 expression.** (A)** Tumors with high GLUT1 expression (16.946±6.661) have higher SUVmax than low expression (8.264±3.177) (*p* < 0.001). **(B)** SUVmax was positively correlated with GLUT1 expression levels (*r^2^* = 0.572, *p* < 0.001). **(C)** Tumors with high HK2 expression (17.960±6.679) have higher SUVmax than low expression (10.031±4.796) (*p* < 0.001). **(D)** SUVmax was positively correlated with HK2 expression levels (*r^2^*= 0.620, *p* < 0.001).

We also used immunohistochemistry to assess the relationship between SUVmax with GLUT1 and HK2 expression. Tumors with high GLUT1 expression have higher SUVmax than low expression (16.946±6.661 vs 8.264±3.177) (Fig. 5A, *p <* 0.001). SUVmax was positively correlated with GLUT1 expression levels (Fig. 5B, *r^2^* = 0.572, *p <* 0.001). Tumors with high HK2 expression have higher SUVmax than low expression (17.960±6.679 vs 10.031±4.796) (Fig. 5C, *p <* 0.001). SUVmax was positively correlated with HK2 expression levels (Fig. 5D, *r^2^* = 0.620, *p <* 0.001). Next, we analyzed the relationship between METTL3 expression with GLUT1 and HK2 expression. The results showed a significant positive correlation between METTL3 expression levels with GLUT1 expression levels (Table 5, *p <* 0.001) and HK2 expression levels (Table 5, *p* = 0.026). These results indicate that METTL3 may affect the uptake of ^18^F-FDG in ESCA by up-regulating GLUT1 and HK2 expression.

## Discussion

Previous studies have shown that overexpression of METTL3 is closely related to gastric cancer, liver cancer and colorectal cancer 16-18. However, the impact of METTL3 on the biological behavior of ESCA has not been reported. To comprehensively investigate the expression of METTL3 in ESCA, the TCGA database was analyzed to identify the overexpression of METTL3 in tumor tissues and its relationship to clinicopathological features. And we found that the METTL3 promoter in ESCA tissues is highly methylated compared to normal esophageal tissue.

In addition, we performed immunohistochemical staining on 57 ESCA patients. The results showed that the positive rates of METTL3 in ESCA tissues and adjacent tissues are 93.0% (53/57) and 8.7% (5/57), respectively. The positive rate of high expression is expected to support METTL3 as a new pathological diagnosis index. The expression of METTL3 in ESCA was significantly higher than that in adjacent tissues, which was consistent with the TCGA database analysis. We also found that overexpression of METTL3 is associated with tumor size and histological differentiation in ESCA patients. Thus, these results suggest that an increase in METTL3 expression may contribute to the development of ESCA, and METTL3 is expected to become a new pathological diagnostic indicator and a potential therapeutic target.

As a new therapeutic target for ESCA, it is important to identify methods that can predict the status of METTL3 expression. Although immunohistochemical staining can do this, this method involves invasive procedures and the availability of tumor tissue is limited. Further development of noninvasive techniques that predict tumor molecular distribution may help to break this limitation. Finally, we analyzed the association between METTL3 expression levels and ^18^F-FDG uptake in ESCA patients and the results showed that the SUVmax of tumors with high METTL3 expression was significantly higher compared to low METTL3 expression. ROC analysis indicated that ^18^F-FDG uptake can predict expression level of METTL3 in ESCA patients. Moreover, multivariate analysis demonstrated a correlation between SUVmax and METTL3 expression. The above results demonstrate that ^18^F-FDG PET/CT scans may help predict the status of METTL3 in ESCA patients. Because METTL3 is generally highly expressed in tumors, a noninvasive method to predict the status of METTL3 and help to monitor the efficacy of METTL3 targeting therapy has important clinical significance.

GLUT1 and HK2 are the rate-limiting enzymes of the Warburg effect, which are closely related to the rate of ^18^F-FDG uptake by ESCA^32^, ^33^. Based on previous studies, we speculate that METTL3 may affect the Warburg effect of tumors^39^-^41^. Our results indicate that SUVmax is positively correlated with the expression of METTL3, and that SUVmax is also positively correlated with the expression of GLUT1 and HK2, which is consistent with previous studies^33^, ^36^. Our results also indicate a positive correlation between METTL3 expression with GLUT1 and HK2 expression. GLUT1, HK2, and SUVmax were significantly higher in high METTL3 expressing tumors compared to low METTL3 expression. These data demonstrate that METTL3 may affect the uptake of ^18^F-FDG in ESCA by up-regulating GLUT1 and HK2 expression.

Our research has some limitations due to the small sample size and retrospective studies. Despite the correlation between METTL3 with GLUT1, HK2, and SUVmax, we need to further confirm our results at the cellular and animal levels. Nonetheless, our findings may be related to noninvasive monitoring of METTL3 expression in ESCA patients.

## Conclusion

The results of immunohistochemistry showed that METTL3 expressed in tumor tissue with high positive rate is expected to become a new pathological diagnosis index and a potential therapeutic target. PET/CT showed an increase in ^18^F-FDG uptake in ESCA patients with high METTL3 expression. Therefore, PET/CT may be a noninvasive monitoring tool for predicting METTL3 expression in ESCA patients. In the absence of *in vitro* tumor tissue, its effect may be more obvious. The expression of METTL3 was positively correlated with the expression of GLUT1 and HK2. METTL3 may increase the uptake of ^18^F-FDG by up-regulating the expression of GLUT1 and HK2. Of course, more prospective studies are needed to confirm our results and to determine if PET/CT can be used to predict the status of METTL3 in ESCA patients. This will help in the decision making of ESCA clinical treatment.

## Figures and Tables

**Table 1:** Characteristics of patients with ESCA (n=57)

Characteristics	No. of patients
**Age (years)**	
Mean ± SD	57.26±7.48
Range	41-77
**Sex**	
Male	43
Female	14
**Pathological type**	
Squamous cell carcinoma	51
Adenocarcinoma	6
**Tumor size (cm)**	
Mean ± SD	3.95±1.85
Range	1.10-9.0
**Histological differentiation**	
Poorly differentiation	25
Moderately-Well differentiation	32
**Tumor staging**	
I-II	41
III-IV	16
**Lymph node metastasis**	
Negative	27
Positive	30
**SUVmax**	
Mean ± SD	14.20±7.05
Range	3.12-30.25

**Table 2:** METTL3 expression in paired tissue specimens from 57 ESCA patients

Specimen	Total	METTL3 expression		*p*
		Negative (%)	Positive (%)	
Tumor tissue	57	4(7.0)	53(93.0)	<0.001
Peritumoral tissue	57	52(91.2)	5(8.7)	

**Table 3:** Relationship between METTL3 expression and the clinicopathological characteristics of ESCA patients

Variable	Total	METTL3 expression		*p*
		Low (%)	High (%)	
**Age (years)**				
<60	35	17(48.6)	18(51.4)	0.366
≥60	22	8(36.4)	14(63.6)	
**Sex**				
Male	43	18(41.9)	25(58.1)	0.594
Female	14	7(50)	7(50)	
Pathological type				
Squamous cell carcinoma	51	23(45.1)	28(54.9)	0.686
Adenocarcinoma	6	2(33.3)	4(66.7)	
**Tumor size (cm)**				
≤3	21	13(61.9)	8(38.1)	0.036
>3	36	12(33.3)	24(66.7)	
**Histological differentiation**				
Poorly differentiation	25	15(60.0)	10(40.0)	0.030
Moderately-Well differentiation	32	10(31.3)	22(68.8)	
**Tumor staging**				
I-II	41	20(48.8)	21(51.2)	0.231
III-IV	16	5(31.3)	11(68.8)	
**Lymph node metastasis**				
Negative	27	13(48.1)	14(51.9)	0.536
Positive	30	12(40.0)	18(60.0)	

**Table 4:** Multivariate analysis of METTL3 expression in 57 ESCA patients

Parameter	Univariate analysis		*p*
	OR	95% CI	
Age (years)	0.612	0.123-3.045	0.549
Sex	0.957	0.195-4.689	0.957
Pathological type	0.563	0.041-7.682	0.666
Tumor size (cm)	0.708	0.126-3.991	0.695
Histological differentiation	2.109	0.500-8.905	0.310
Tumor staging	0.961	0.135-6.828	0.968
Lymph node metastasis	0.691	0.123-3.891	0.675
SUVmax	1.316	1.110-1.560	0.002

OR: Odds ratio. 95% CI: 95% Confidence interval.

**Table 5:** Relationship between METTL3 with GLUT1 and HK2 expression

		METTL3 expression		*p*
		Low	High	
GLUT1 expression	Low	14	4	<0.001
	High	11	28	
HK2 expression	Low	16	11	0.026

## Abbreviations

ESCA: Esophageal carcinoma
m6A: N6-methyladenine
METTL3: Methyltransferase 3
PET/CT: Positron emission tomography/computed tomography
^18^F-FDG: ^18^F-fluorodeoxyglucose
SUVmax: standard uptake value
GLUT1: Glucose transporter 1
HK2: hexokinase 2
TCGA: The Cancer Genome Atlas
ROI: region of interest
TBST: Tris-Buffered Saline Tween-20
SD: standard deviation
ROC: receiver operating characteristic
